# Latest Update on Prevention of Acute Chemotherapy-Induced Nausea and Vomiting in Pediatric Cancer Patients

**DOI:** 10.1007/s11912-019-0840-0

**Published:** 2019-08-15

**Authors:** Farha Sherani, Catherine Boston, Nkechi Mba

**Affiliations:** Department of Pediatric Hematology & Oncology, Cancer & Blood Disorders Center, Driscoll Children’s Hospital, 3533 S. Alameda St, Corpus Christi, TX 78411 USA

**Keywords:** Nausea, Vomiting, Antiemetic, Chemotherapy-induced nausea and vomiting, CINV, Supportive care, Guidelines

## Abstract

**Purpose of Review:**

Chemotherapy-induced nausea and vomiting (CINV) is a common cause of acute morbidity that impacts quality of life in children receiving cancer treatment. Here, we review the evolution of CINV prophylaxis guidelines in children, with an emphasis on the literature published in the last 5 years, to bring the reader up to date.

**Recent Findings:**

Recent studies have led to the adoption of the “triple therapy” regimen of antiemetic prophylaxis (a 5-HT3 antagonist, dexamethasone, and a neurokinin-1 antagonist) as the backbone of recommendations for the prevention of CINV in children. Areas of new data include the addition of aprepitant and inclusion of palonosetron as a non-inferior 5-HT3 antagonist. In addition, there are emerging pediatric data informing patient-derived risk factors associated with CINV risk and classification of antineoplastic drugs based on emetogenicity.

**Summary:**

Several recent pediatric studies have shaped published guidelines for CINV prophylaxis in children.

## Introduction

Chemotherapy-induced nausea and vomiting (CINV) remains one of the most common and distressing adverse effects of chemotherapy in children receiving cancer treatment [1]. It affects quality of life during treatment for both patient and caregiver. Historically, there have been few data on the prevalence in pediatrics and few guidelines on prophylaxis of CINV even as recently as 10 years ago. Prior to validation of patient self-report tools in this population like the Pediatric Nausea Assessment Tool (PeNAT) and pictorial scales [2, 3], prophylaxis was based largely on extrapolation from adult data and expert opinion. These tools have directly impacted the feasibility of conducting pediatric-specific studies.

Two major changes have been outlined in recent guidelines, namely the addition of aprepitant and the inclusion of palonosetron which will be discussed here in detail. The most comprehensive clinical guidelines for pediatrics come as a four-part series published by the Pediatric Oncology Group of Ontario (POGO), a collaboration of the five pediatric oncology programs in Ontario, Canada [4, 5••, 6, 7••, 8, 9]. These guidelines also include recommendations for treatment of breakthrough and anticipatory nausea and vomiting, adjuvant non-pharmacologic therapies, and dosing of antiemetic agents. They have cumulatively become the framework for many institutional CINV guidelines and are the endorsed supportive care guidelines for the Children’s Oncology Group [10•].

Here, we highlight key principles that inform the background for the main recommendations in these guidelines, including classification of drugs by emetogenicity risk and reviewing what has been studied about risk factors associated with CINV. We then review the evolution of CINV data and prophylaxis regimens in children receiving antineoplastic drugs.

## Classification of Drugs by Emetogenicity

The inherent emetogenicity of a drug regimen is the basis of current guidelines addressing CINV prophylaxis in children. Cancer drugs are classified as either minimal, low, medium, or highly emetogenic risk, based on the incidence of vomiting without any antiemetic prophylaxis. Minimal emetogenic risk is assigned to drugs that result in < 10% of patients vomiting after intravenous administration of that drug. Low risk is assigned to 10–30%, moderate risk to 31–90%, and high risk to > 90% incidence of vomiting.

The parameters are set to pertain to the acute phase of vomiting in chemotherapy naïve patients, defined as occurring < 24 h from drug administration. In the case of multi-agent chemotherapy administration, the drug with the highest emetogenicity drives the emetogenicity risk of that combination. When antiemetic prophylaxis is given, >/= 10% incidence in emesis is defined as failure of prophylaxis requiring an update in chemotherapy emetogenicity risk classification.

Nausea is excluded due to the sparsity of pediatric data using a validated measurement tool for nausea. Importantly, there has been a large shift to using pediatric-specific data for the current version of this POGO guideline [5•] from its previous publication [4]. It classifies 49-single agent and 13-combination agent chemotherapy regimens into the four previously defined categories by emetogenicity: minimal, low, moderate, or highly emetogenic chemotherapy (Table 1). The publication is not a simple update of its previous counterpart (which was an adaptation of adult guidelines); it is rather a de novo guideline based on evidence from pediatric oncology patients. As a result, it classifies fewer antineoplastic drugs than its predecessor.

**Table 1:** Emetogenic Risk Classification of Antineoplastic Drugs as per the latest POGO guidelines in 2019. Reproduced from Paw Cho Sing, E, Robinson, PD, Flank, J, et al. Classification of the acute emetogenicity of chemotherapy in pediatric patients: a clinical practice guideline. Pediatr Blood Cancer. 2019; 66:e27646, with permission from John Wiley and Sons

Minimal risk (< 10%)	Low risk (10–30%)	Moderate risk (30–90%)	High risk (> 90%)
Single-agent regimens: • Asparaginase (*E. coli*) IM ≤ 6000 IU/m2 • Asparaginase (Erwinia) IM ≤ 25 000 IU/m2 • Chlorambucil ≤ 0.2 mg/kg/day PO • Doxorubicin IV 10 mg/m2 • Liposomal doxorubicin IV ≤ 50 mg/m2 • Mercaptopurine PO ≤ 4.2 mg/kg • Methotrexate PO/SC ≤ 10 mg/m2 • Pracinostat 25–45 mg/m2/dose PO • Vincristine IV ≤ 1.5 mg/m2	Single-agent regimens: • Cyclophosphamide IV 500 mg/m2 • Cyclophosphamide PO 2–3 mg/kg • Dasatinib PO 60–120 mg/m2 • Erlotinib PO 35–150 mg/m2/day • Everolimus PO 0.8–9 mg/m2/day • Gefitinib PO 150–500 mg/m2/day • Imatinib PO 260 mg/m2/day • Mafosfamide IT 1–6.5 mg • Melphalan PO 0.2 mg/kg • Mercaptopurine PO ≤ 4.2 mg/kg • Methotrexate 38–83 mg/m2 IV • Mitoxantrone IV ≤ 33 mg/m2 • Procarbazine PO 50–100 mg/m2/day • Ruxolitinib PO 15–21 mg/m2 • Selumetinib PO 20–30 mg/m2 • Sorafenib PO 150–325 mg/m2 • Temozolomide PO 200 mg/m2	Single-agent regimens: • Cyclophosphamide IV 1000 mg/m2 • Cytarabine IV 75 mg/m2 • Dactinomycin IV 10 μg/kg • Doxorubicin IV 25 mg/m2 • Gemtuzumab IV 3–9 mg/m2 • Imatinib PO > 260 mg/m2/day • Interferon alpha IV 15–30 million U/m2/day • Ixabepilone IV 3–10 mg/m2 • Methotrexate IV 5 g/m2 • Methotrexate IT • Topotecan PO 0.4–2.3 mg/m2/day	Single-agent regimens: • Asparaginase (Erwinia) IV ≥ 20 000 IU/m2 • Busulfan IV ≥ 0.8 mg/kg • Busulfan PO ≥ 1 mg/kg • Carboplatin IV ≥ 175 mg/m2 • Cisplatin IV ≥ 12 mg/m2 • Cyclophosphamide IV ≥ 1200 mg/m2 • Cytarabine IV ≥ 3 g/m2/day • Dactinomycin IV ≥ 1.35 mg/m2 • Doxorubicin IV ≥ 30 mg/m2 • Idarubicin PO ≥ 30 mg/m2 • Melphalan IV • Methotrexate IV ≥ 12 g/m2
Multiple-agent regimens: • Cisplatin ≤ 60 mg/m2 intra-arterially + doxorubicin ≤ 30 mg/m2/dose intra-arterially • Cisplatin ≤ 60 mg/m2 intra-arterially + pirarubicin ≤ 30 mg/m2 intra-arterially • Mercaptopurine PO ≤ 2.5 mg/kg + methotrexate PO ≤ 0.1 mg/kg/day	Multiple-agent regimens: • Cytarabine IV 60 mg/m2 + Methotrexate IV 90 mg/m2	Multiple-agent regimens: • Cytarabine IV 100 mg/m2/dose + daunorubicin IV 45 mg/m2 + Etoposide IV 100 mg/m2 + prednisolone PO + thioguanine PO 80 mg/m2 • Cytarabine 60 or 90 mg/m2 + methotrexate 120 mg/m2 • Liposomal doxorubicin IV 20–50 mg/m2 + topotecan PO 0.6 mg/m2/day	Multiple-agent regimens: • Cyclophosphamide > 600 mg/m2 + dactinomycin >/= 1 mg/m2 • Cyclophosphamide ≥ 400 mg/m2/dose + doxorubicin ≥ 40 mg/m2 • Cytarabine ≥ 90 mg/m2/dose IV + methotrexate IV ≥ 150 mg/m2 • Cytarabine IV + teniposide IV • Dacarbazine ≥ 250 mg/m2/dose IV + doxorubicin IV ≥ 60 mg/m2 • Dactinomycin 900 μg/m2/dose IV + ifosfamide 3 g/m2 • Etoposide IV ≥ 60 mg/m2/dose + ifosfamide IV ≥ 1.2 g/m2 • Etoposide IV ≥ 250 mg/m2/dose + thiotepa IV ≥ 300 mg/m2

*All drugs are listed per dose and for intravenous administration unless otherwise stated

## Emetic Clinical Syndromes

In addition to classifying drug regimens by inherent emetogenicity, the recognition of distinct emetic clinical syndromes also contributed to the major advances made in CINV control. For example, delayed-phase CINV is now well defined and was originally most comprehensively studied in cisplatin. Briefly, cisplatin causes immediate nausea and vomiting peak within 1–2 h of administration, emesis subsiding at around 24 h, then a second peak of nausea and vomiting at 48–72 h [11]. Other agents that can cause delayed vomiting include cyclophosphamide, anthracyclines, and carboplatin [12]. Based on the cisplatin model, acute-phase vomiting became defined as < 24 h following administration of the antineoplastic drug and delayed-phase vomiting as > 24 h [13]. Recognition of emetic clinical syndromes such as delayed-phase CINV associated with a drug must be taken into account when interpreting the current guidelines, as they have been only written for acute vomiting in chemotherapy-naïve patients.

Anticipatory nausea is a clinical emetic syndrome that also contributes to a patient’s emetic risk. It is defined as the conditioned response to an initial emetic experience. It has been shown that high levels of CINV at a preceding emetic experience are correlated with high anticipatory nausea at subsequent encounters [14]. Understanding anticipatory nausea has underscored the importance of optimal CINV control and helped achieve better control for CINV in patients receiving antineoplastic therapy over the past three decades.

## Risk Factors Associated with CINV

In addition to the treatment-related factors such as dose and inherent emetogenicity of chemotherapy drugs, several patient-related factors have been shown to be associated with risk of emetogenicity in adults [12, 13, 15]. These include age, sex, and high pretreatment expectation of nausea, among others. In addition, anticipatory nausea as mentioned above is unique to each patient based on their prior CINV experience and plays a factor in the “total” risk of emetogenicity with each subsequent encounter.

In pediatrics, there has been only one recent study on this topic [16] and is a secondary analysis of a previously published multicenter, international, prospective, randomized, single-blind trial. It was initially evaluating the efficacy of acupressure in antiemetic prophylaxis regimens for children receiving highly emetogenic chemotherapy; nausea severity scored by PeNAT was then analyzed by a proportional odds generalized estimating equation approach and reported as acute or delayed phase CINV. It showed acute-phase CINV was associated with non-white race. In addition, delayed-phase CINV was associated with poor acute-phase CINV control, non-CNS cancer, and administration of cisplatin. It is also one of few studies using a validated nausea tool in pediatrics. Further work in this direction is needed to evaluate the potential of personalizing antiemetic prophylaxis to include patient-related risk factors in children.

## The Evolution of Clinical Practice Guidelines for Prevention of CINV in Pediatrics

Prevention and management of CINV as an important supportive care goal came to the forefront in the late 1990s in adult medicine. This was based on multiple investigations that improved our understanding of the pathophysiology of chemotherapy-induced nausea, and specifically, the subsequent introduction of the 5-HT3 antagonists. Here, we first review the main classes of antiemetic drugs included in national CINV prevention guidelines.

### 5-HT3 Antagonists

There are several neurotransmitters involved in the pathophysiology of CINV, however, 5-HT (serotonin) has been shown to be the most important to date [17, 18]. In addition, of all the 5-HT receptors, selective antagonists of the 5-HT3 receptors are currently the single most effective class of antiemetics available for acute-phase CINV. There are five widely available 5-HT3 selective antagonists: ondansetron, granisetron, dolasetron, tropisetron, and a more recently introduced drug, palonosetron. Ondansetron was the first to be US Food and Drug Administration (FDA) approved for adults in 1991, palonosetron was the most recent. 5-HT3 selective antagonists act on receptors present in both central nervous system targets such as the area postrema, and the peripheral nervous system such as vagal afferents in the intestine. They are a well-tolerated class of drugs, with no limiting toxicity at typical doses. The most common adverse events include headache and constipation. In addition, the oral and intravenous administrations are therapeutically equivalent.

5-HT3 antagonists form the cornerstone of antiemetic prophylaxis regimens for moderate to highly emetogenic chemotherapy. Several randomized controlled trials and meta-analyses in adults, and then in children, have shown their efficacy [19–21]. Tricco et al. [21] systemically reviewed over 299 studies with 58,412 patients, published 1995–2015. Twenty-five (8%) of those studies included pediatric-only trials and eight (2.7%) included combined adult and pediatric patients. Primary outcomes were to evaluate both efficacy and safety when using 5-HT3 antagonists alone and with dexamethasone. No statistically significant differences in the overall effectiveness between the 5-HT3 antagonists were observed. Combination of a 5-HT3 antagonist with dexamethasone provided superior antiemetic control than a 5-HT3 antagonist alone. None of the studies compared treatment versus placebo arm for adverse effects. However, when specifically evaluating for QTc prolongation, a known adverse effect of the 5-HT3 antagonists, a network meta-analysis showed a slightly increased risk of prolongation of QTc with the combination of dolasetron and dexamethasone versus ondansetron and dexamethasone. The analysis included four randomized controlled trials reflecting 3358 adults and children, odd ratio of 2.94 and 95% CI 2.13–4.17. Of note, all of the 5-HT3 antagonists come with labeling of possible QTc prolongation, however, in children, the risk is minimal given single doses of > 16 mg are never used and co-morbidities that increase this risk (use of antiarrhythmic, cardiovascular disease) are much lower. In fact, even when studies have found statistically significant minor EKG changes, none have reported dangerous rhythm disturbances or adverse events in children [22].

### Corticosteroids

Corticosteroids have been used for their antiemetic properties in adults since the 1980s, although their mechanism of action remains unknown. The most commonly used corticosteroids are dexamethasone and methylprednisolone. Often, they are used as single-agent therapy for low emetogenic chemotherapy, and in combination with 5-HT3 (± neurokinin-1 antagonists) for moderate to highly emetogenic chemotherapy [7••, 23–26]. A major advantage of corticosteroids is that they provide antiemetic control for all nausea, acute and delayed emesis [27–29]. A recent randomized double-blind study in adults compared a combination of palonosetron (a long-acting 5-HT3 antagonist), dexamethasone and aprepitant (n neurokinin-1 antagonist) given on day 1, then compared groups receiving either dexamethasone or aprepitant for days 2–3 for CINV control [29]. They concluded dexamethasone and aprepitant provided equivalent efficacy (and toxicity) in that particular regimen. Corticosteroids are generally well tolerated at antiemetic doses; most common adverse effects include steroid-induced acne, increased appetite, insomnia, and gastrointestinal symptoms.

### Neurokinin-1 Antagonists

Neurokinin is a member of a group of proteins called tachykinins that have multiple regulatory functions. Specifically, neurokinin-1 receptors are present diffusely in the central nervous system including the area postrema and have peripheral targets in the gastrointestinal system as well [30, 31]. Neurokinin-1 antagonists are the newest class of antiemetic agents to gain acceptance in adult and pediatric CINV guidelines. Aprepitant, a potent and selective oral neurokinin-1 antagonist, was the first in its class to be FDA approved in adults in 2003. There is robust evidence for the efficacy of neurokinin-1 antagonists in preventing CINV, especially delayed onset nausea and vomiting [32–35]. Although the mechanism has not been entirely deduced, it is thought to act on both its central and peripheral targets for full effect. Of note, it is a moderate P-450 3A4 inhibitor, and therefore when administered with corticosteroids where the latter is being administered for antiemetic effect, the dose of the steroid must be reduced by 50% [36]. The exception is when corticosteroids are being administered as antineoplastic chemotherapy. Several studies have looked at drug-drug interactions, as aprepitant is also a weak CYP 2C9 inducer. A systematic review by Patel et al. [37] looked at literature up until September 2016 and identified 64 publications. They reviewed evidence for drug-drug interactions and adverse effects for some commonly co-administered drugs, citing that these interactions must be reviewed prior to using aprepitant (see Table 2). Notable interactions include increased neurotoxicity with ifosfamide and increased efficacy of oxycodone.

**Table 2:** Aprepitant drug-drug interactions and adverse effects for some commonly co-administered drugs

Significant pharmacokinetic drug interactions with aprepitant/fosaprepitant reported	• Bosutinib PO • Cabazitaxel IV • Cyclophosphamide IV • Dexamethasone PO • Methylprednisolone IV • Midazolam PO/IV • Oxycodone PO • Tolbutamide PO
Possible adverse events resulting from an interaction with aprepitant/fosaprepitant	• Alcohol (impaired cognition) • Anthracyclines (infusion via the same peripheral vein may cause a local reaction at infusion site) • Ifosfamide (neurotoxicity) • Oxycodone (increased feeling of a “high,” decreased respiratory rate) • Quetiapine (somnolence) • Selective serotonin reuptake inhibitors/serotonin-norepinephrine reuptake inhibitors (vomiting) • Warfarin (INR changes)

## Triple Therapy for Moderate and Highly Emetogenic Chemotherapy

The main pharmacologic classes of drugs with a high therapeutic index in CINV prophylaxis as described above are the 5-HT3 antagonists, corticosteroids, and neurokinin-1 antagonists. All three have different mechanisms of actions. Their combination has been shown to provide maximum antiemetic control, particularly for patients undergoing highly emetogenic chemotherapy. It has become the most widely accepted antiemetic prophylaxis backbone; often referred to as a “triple therapy” antiemetic regimen.

Antiemetic guidelines have been published by several large cancer organizations, including the American Society of Clinical Oncology (ASCO), the National Comprehensive Cancer Network (NCCN), European Society of Medical Oncology (ESMO), and the Multinational Association of Supportive Care in Cancer (MASCC) [38–41]. All four groups published these guidelines in adults in 2006–2007, with broad agreement and largely based on the “triple therapy” antiemetic regimen as a backbone for moderate to highly emetogenic chemotherapy.

In children, the main professional group to publish CINV prophylaxis guidelines is the Pediatric Oncology Group of Ontario (POGO), a collaboration of the five pediatric oncology programs in Ontario, Canada. In 2013, they published a guideline that was extrapolated from the adult versions [6]. However, due to lack of safety and efficacy data in pediatrics, the “triple therapy” regimen was restricted to children >/= 12 years of age receiving highly emetogenic chemotherapy. The class of antiemetic drugs with the least amount of pediatric data was the neurokinin-1 antagonists. Therefore, for children under 12 years of age, only a 5-HT3 antagonist and dexamethasone was recommended as prophylaxis, even for highly emetogenic chemotherapy, a regimen typical for drugs classified as moderately emetogenic risk.

Since then, there has been a revolution of pediatric-based data in the field of CINV prophylaxis. The most recent Pediatric Oncology Group of Ontario (POGO) recommendations are now a comprehensive, four-part series of guidelines that include recommendations for CINV prophylaxis, treatment of breakthrough and anticipatory nausea and vomiting, adjuvant non-pharmacologic therapies, and dosing of antiemetic agents [4, 5••, 7••, 8, 9]. They have cumulatively become the framework for many institutional CINV guidelines and are the endorsed supportive care guidelines for the Children’s Oncology Group [10•]. There are two major differences in the antiemetic prophylaxis regimen recommendations from its earlier version in 2013, which will be discussed next.

## Recent Changes to Antiemetic Prophylaxis Regimen Guidelines in Children

### Addition of Aprepitant to Antiemetic Regimen

To address the lack of safety and efficacy data in children with neurokinin-1 antagonists, two randomized, double-blind, placebo-controlled studies were published in 2015. Bakshi et al. [42] evaluated 93 children, aged 5–18 years, undergoing highly emetogenic chemotherapy. Kang et al. [43] evaluated 302 patients at 49 sites in 24 countries, undergoing moderate or highly emetogenic chemotherapy. Enrolled patients included 6 months to 17 years old; less than 12-year-old children were dosed with aprepitant 3 mg/kg on day 1 up to 125 mg, 2 mg/kg on days 2–3 up to 80 mg. Some limitations include no pharmacokinetic data to guarantee therapeutic levels. However, neither trial showed any increased toxicity attributable to aprepitant use. In addition, both studies showed a statistically significant improvement in CINV control with aprepitant, in both acute and delayed phases. These studies are reflected in the expansion of the recommendation to add aprepitant for children receiving highly emetogenic chemotherapy to >/= 6 months of age, previously limited to children 12 years and older [7••].

### Inclusion of Palonosetron to the 5-HT3 Antagonists

Palonosetron is a newer 5HT3 antagonist with a longer half-life than ondansetron which has been shown to be effective in preventing CINV in adults. It can be dosed every 72 h as opposed to every 8 h, allowing fewer doses to be administered to achieve antiemetic effect. A recent, randomized non-inferiority study in pediatric patients performed by Kovacs et al. showed that palonosetron was non-inferior to ondansetron as prevention of CINV in children receiving moderately and highly emetogenic chemotherapy. Furthermore, a dose of palonosetron 0.2 mg/kg was superior to both palonosetron 0.1 mg/kg and ondansetron in preventing acute, delayed, and overall CINV [44•]. This and other non-inferiority studies led to the FDA approval of this medication in 2014 for children ages 1 month to 17 years, which marked the first antiemetic approved for children ages 1–6 months. In addition, palonosetron has also shown to significantly decrease delayed (24–120 h after first chemotherapy) CINV due to moderately and highly emetogenic chemotherapy regimens (*p* = 0.004), which may make it a first choice for chemotherapy regimens > 3 days [45]. In resource-poor countries, it is more cost-effective than ondansetron without sacrificing control of nausea and vomiting. There is also no significant difference in the adverse effect profiles of palonosetron compared to ondansetron [46]. This has led to the recent update of the Pediatric Oncology Group of Ontario’s 2013 Guideline for the Prevention of Acute Nausea and Vomiting due to Antineoplastic Medication in Pediatric Cancer Patients to include palonosetron as an alternate 5HT3 antagonist in combination with dexamethasone for moderately emetogenic chemotherapy, and to dexamethasone and aprepitant for highly emetogenic chemotherapy, allowing palonosetron to become part of “triple therapy” for the prevention of pediatric CINV prophylaxis [7••].

## Antiemetic Drugs with a Lower Therapeutic Index

While other pharmacologic therapies have been tried for the prevention of pediatric CINV, they lack the robust pediatric clinical data to make them standard of care. However, two commonly used drugs for their antiemetic potential were recently investigated in the pediatric setting, lorazepam and nabilone. A pediatric study testing the addition of lorazepam to granisetron in children with acute lymphoblastic leukemia showed no difference in CINV in the group compared to the group without lorazepam [47]. A pediatric retrospective study of the cannabinoid, nabilone, showed a 56% decrease in CIV when combined with several other antiemetic regimens, however, this was similar to the rate of CIV control using 5-HT3 antagonists alone. Furthermore, nabilone had to be discontinued in 10 patients and decreased in 5 patients due to adverse events, most common being sedation. The adverse effects and the fact that this was a retrospective study that did not standardize the antiemetic regimen prohibits the recommendation of adding this routinely to pediatric CINV prophylaxis [48]. There are no robust pediatric clinical trials recommending promethazine or metoclopramide for routine use in children. Due to extrapyramidal effects in young children with metoclopramide, it is recommended not to use it in children < 1 year, and limit use to 5 days in children < 5 years [49].

## Integrative Medicine Options for CINV Prophylaxis in Children

Non-pharmacologic integrative options are an attractive area of practice since they may be a useful adjunct to standard medications in CINV control. In addition, they are more likely to be without side effects than pharmacologic therapies and add other benefits including therapeutic intention, perceived stress relief, and patient and caregiver enjoyment. Many therapies are free or inexpensive making them a convenient adjunct to pharmacologic therapy, however, there are no recent robust pediatric studies to recommend the use of them. In a study of oral ginger versus placebo added to dexamethasone and ondansetron for patients receiving highly emetogenic chemotherapy for bone sarcoma, there was an improvement in both acute and delayed nausea and vomiting in the group receiving ginger [50]. In a small pediatric double-blind study of ginger aromatherapy for the prevention of chemotherapy-induced nausea (CIN), there was no difference observed among patients who did and did not receive aromatherapy. However, it is one of few studies to have used a standardized nausea tool, the PeNAT [51]. Ginger aromatherapy was reported to be well-tolerated and enjoyed by patients and caregivers alike. The recent POGO guidelines for pediatric CINV have a weak recommendation for therapies such as acupuncture, acupressure, guided imagery, music therapy, virtual reality, progressive muscle relaxation, and psycho-educational support [7••], mostly due to lack of evidence.

## Conclusion

Several recent pediatric studies have shaped published guidelines for CINV prophylaxis in children. Use of combination therapy with a backbone of 5-HT3 antagonists, dexamethasone, and neurokinin-1 antagonists have been proven to provide improved antiemetic control for both acute and delayed phases (Table 3).

**Table 3:** Recommended antiemetic regimen for single-day chemotherapy

Emetogenic risk	Antiemetic regimen				
	> 6 months of age, chemotherapy NOT known to interact with aprepitant	> 6 months of age, chemotherapy KNOWN to interact with aprepitant*	> 6 months of age, chemotherapy NOT known to interact with aprepitant and who cannot receive dexamethasone^%^	< 6 months old	< 6 months old who cannot receive dexamethasone^%^ OR > 6 months old and cannot receive aprepitant or dexamethasone^%^
High	Granisetron, ondansetron, or palonosetron + dexamethasone + aprepitant	Granisetron, ondansetron, or palonosetron + dexamethasone	Palonosetron^#^ + aprepitant	Granisetron, ondansetron or palonosetron + Dexamethasone	Palonosetron
Moderate	No restrictions	> 6 months of age, who cannot receive dexamethasone^%^	> 6 months of age, chemotherapy KNOWN to interact with aprepitant* and cannot receive dexamethasone^%^		< 6 months old who cannot receive dexamethasone^%^
	Granisetron, ondansetron, or palonosetron + dexamethasone	Granisetron, ondansetron or, palonosetron + Aprepitant	Palonosetron		Palonosetron
Low	Ondansetron or granisetron				
Minimal	No routine prophylaxis				

*Chemotherapy agents known to interact with aprepitant: Ifosfamide

^%^Diseases for which dexamethasone may NOT be used as an anti-emetic agent: any leukemia or lymphoma, any histiocytosis and other diseases in which corticosteroids are used as antineoplastic therapy

We recently adopted the Children’s Oncology Group CINV prophylaxis guidelines [10•] at our institution, specifically by expanding our use of double and triple antiemetic therapy, for moderate and highly emetogenic chemotherapy respectively. We have observed unanimously better CINV control, less therapy-related malnutrition in patients undergoing intense chemotherapy regimens, and are able to maintain > 95% adherence rates. It has been feasible to use in both outpatient and inpatient settings.

Despite the progress that CINV prophylaxis has made in the last two decades, there are a number of areas that still lack any or at least robust, pediatric-specific data. There are still several antineoplastic drugs with little pediatric-based data on the emetogenicity risk (Table 1) and little data on how best to manage multi-day chemotherapy administrations. In addition, given the increasing preferences for integrative medicine among patients and their families, studies on complementary and alternative therapies for CINV need to be done. Well-controlled and accurately powered trials are lacking in this increasingly popular area. With relatively new pediatric nausea scales now available, such as the Pediatric Nausea Assessment Tool (PeNAT) and pictorial scales [2, 3], all future studies should also incorporate preventing nausea as a primary goal.

With diligent adherence to evidence-based antiemetic guidelines, it is possible to drastically reduce the incidence of one of the most feared and problematic adverse effects of antineoplastic therapy in children. The most recent changes based on randomized controlled trials in the area have allowed the expansion of this optimal control to patients >/= 6 months of age.
